# The Effect of Size and Asymmetry at Birth on Brain Injury and Neurodevelopmental Outcomes in Congenital Heart Disease

**DOI:** 10.1007/s00246-021-02798-5

**Published:** 2021-12-01

**Authors:** Shalin A. Parekh, Stephany M. Cox, A. James Barkovich, Vann Chau, Martina A. Steurer, Duan Xu, Steven P. Miller, Patrick S. McQuillen, Shabnam Peyvandi

**Affiliations:** 1 Division of Cardiology, Department of Pediatrics, Benioff Children’s Hospital, University of California, Mission Hall Box 0544, 550 16th Street, 5th Floor, San Francisco, CA 94158 USA; 2 Division of Developmental Pediatrics and Cardiology, Department of Pediatrics, Benioff Children’s Hospital, University of California, San Francisco, USA; 3 Department of Radiology, University of California, San Francisco, USA; 4 Department of Neurology, Hospital for Sick Children, University of Toronto, Toronto, ON Canada; 5 Division of Critical Care, Department of Pediatrics, Benioff Children’s Hospital, University of California, San Francisco, USA

**Keywords:** Birth asymmetry, Birth anthropometry, Brain injury, Neurodevelopment, Congenital heart disease

## Abstract

Poor and asymmetric fetal growth have been associated with neonatal brain injury (BI) and worse neurodevelopmental outcomes (NDO) in the growth-restricted population due to placental insufficiency. We tested the hypothesis that postnatal markers of fetal growth (birthweight (BW), head circumference (HC), and head to body symmetry) are associated with preoperative white matter injury (WMI) and NDO in infants with single ventricle physiology (SVP) and d-transposition of great arteries (TGA). 173 term newborns (106 TGA; 67 SVP) at two sites had pre-operative brain MRI to assess for WMI and measures of microstructural brain development. NDO was assessed at 30 months with the Bayley Scale of Infant Development-II (*n* = 69). We tested the association between growth parameters at birth with the primary outcome of WMI on the pre-operative brain MRI. Secondary outcomes included measures of NDO. Newborns with TGA were more likely to have growth asymmetry with smaller heads relative to weight while SVP newborns were symmetrically small. There was no association between BW, HC or asymmetry and WMI on preoperative brain MRI or with measures of microstructural brain development. Similarly, growth parameters at birth were not associated with NDO at 30 months. In a multivariable model only cardiac lesion and site were associated with NDO. Unlike other high-risk infant populations, postnatal markers of fetal growth including head to body asymmetry that is common in TGA is not associated with brain injury or NDO. Lesion type appears to play a more important role in NDO in CHD.

**Supplementary Information:**

The online version contains supplementary material available at 10.1007/s00246-021-02798-5.

## Background

Infants with complex congenital heart disease (CHD) have evidence of brain immaturity, are at increased risk for neonatal brain injury and subsequent adverse neurodevelopmental (ND) outcomes [1, 2]. Among patients with CHD, postnatal nutrition and growth appear to influence ND outcomes [3–7]; however, the relationship between fetal growth and neurodevelopment is unclear [6]. Newborns with complex CHD are known to have abnormalities of somatic growth beginning in utero. Infants with single ventricle physiology (SVP) have lower birth weights (BW) and smaller head circumference (HC) compared to normal infants at a similar gestational age. Interestingly, those with transposition of the great arteries (TGA) tend to exhibit asymmetric growth, with normal BW though a smaller HC, compared to the general population of normal infants [8–10], likely due to aberrant fetal circulatory flow patterns affecting the brain [11–13].

In other neonatal populations, such as those with intrauterine growth restriction and prematurity, a slower rate of increase in prenatal HC and smaller HC at birth have both been associated with worse ND outcomes [14–17]. Studies on these populations of infants have also revealed associations between BW and risk of neonatal white matter injury [18, 19], with a similar pattern of injury seen in CHD infants [20, 21]. Asymmetric growth, a common finding in growth-restricted infants with “relative brain-sparing”, has also been linked to poor neuromotor and behavioral outcomes within the first year of life [22–24]. However, in the CHD population, the relationship of fetal growth, size and symmetry at birth with developmental outcomes is less clear. While some studies have suggested a link between head circumference at birth and ND outcomes [4], others have suggested no association between fetal growth or neonatal birth anthropometric parameters and ND outcomes among patients with critical CHD (e.g., hypoplastic left heart syndrome and transposition of the great arteries) [25].

Our primary aim was to study the association between postnatal markers of fetal growth (weight, length, HC, and head to bodyweight asymmetry at birth) with preoperative brain white matter injury and neonatal brain development. We also sought to understand the relationship between parameters of birth size and ND outcomes at 30 months as a secondary aim. We *hypothesize* that, similar to other patient populations like preterm and growth-restricted infants, fetal growth restriction and, specifically, growth asymmetry may explain some of the unexplained variation noted in ND outcomes in two specific forms of CHD, transposition of the great arteries (TGA) and single ventricle physiology (SVP), with varied fetal growth patterns.

## Methods

This is a secondary data analysis of a prospective cohort study. Between 2001 and 2019, newborns with critical CHD at the University of California-San Francisco Benioff Children’s Hospital (UCSF) and University of British Columbia (UBC) participated in a prospective protocol obtaining pre-operative and post-operative magnetic resonance imaging (MRI) and ND evaluation. [1, 21]. The MRI protocols at both sites are described in the supplement. Patients diagnosed as having TGA or SVP were included in the present study. *TGA was defined as* great vessel malposition with the aorta arising from the right ventricle and pulmonary artery arising from the left ventricle with or without a ventricular septal defect. *SVP was defined as* the absence of 1 of 2 functioning ventricles requiring a palliative surgical intervention for survival in the newborn period.

Patients were excluded from the study if they were born before 37 weeks’ gestation; were born with a suspected congenital infection; had clinical evidence of a congenital malformation or syndrome; or had a suspected or confirmed genetic or chromosomal anomaly. The pre-operative brain MRI was obtained as soon as was safely feasible after birth; the post-operative MRI was obtained prior to hospital discharge. The average separation between pre- and post-operative brain MRI was 15 days. This study was approved by the institutional review board at each site. Informed consent was obtained from the parents for study participation.

The primary predictors for this study were anthropometric measures at birth including body weight, HC, body length, and asymmetry of head to body weight at birth. *Z* scores were calculated based on sex and gestational age from the Fenton’s preterm infant growth chart [26, 27]. Asymmetry was calculated as the difference between weight *Z* score and head circumference *Z* score (BWz-HCz). We defined infants with BWz-HCz ≥ − 0.5 and ≤  + 0.5 as symmetric. Among the asymmetric infants those with BWz-HCz < − 0.5 were defined as head sparing while those with BWz-HCz >  + 0.5 were defined as non-head-sparing asymmetry.

The primary outcome for this study was moderate-to-severe white matter injury (WMI) on the pre-operative MRI. WMI was graded by a neuroradiologist at each site as none to mild (< 3 foci each < 2 mm) or moderate-to-severe (> 3 foci or any foci > 2 mm) as previously described [21]. The neuroradiologists were blinded to clinical variables. To incorporate other forms of injury seen in this population, the Brain Injury Severity (BIS) score was also described as follows: 0 as none to mild WMI, 1 as stroke, and 2 as moderate-to-severe WMI [28, 29]. Post-natal brain development was assessed as the change in brain white matter fractional anisotropy between pre- and post-operative brain MRI. With increasing microstructural brain development, fractional anisotropy (FA) increases [30]. FA from 5 different regions of the brain (1) anterior white matter; (2) central white matter; (3) posterior white matter; (4) posterior limb of the internal capsule; (5) optic radiations were measured in 1 cm cubic voxels and averaged for each MRI scan [1, 29]. The process for measuring fractional anisotropy and the anatomical regions for which this was measured is as described [1, 29].

The secondary outcome for this study was ND outcome based on the Bayley Scales of Infant Development-II (BSID-II), which was available for a subset of the study population earlier in the cohort. This was administered by a psychologist (blinded to the cardiac diagnosis) at 30 months of age to obtain the Psychomotor Development Index (PDI) and the Mental Development Index (MDI) at each of the different study sites.

### Statistical Analysis

Clinical characteristics between infants with none/mild WMI and moderate/severe WMI were compared using chi-squared or Fisher’s exact test for categorical variables and Student’s *t* test for normally distributed continuous variables. Univariate linear regression analysis was used to assess the association of our primary predictor and other clinical factors with MDI and PDI scores at 30 months of age. Multiple linear regression model was used to adjust for site at which participants were enrolled (UCSF vs UBC) and other relevant clinical variables.

Change in FA between pre- and postoperative brain MRIs was analyzed using mixed models for longitudinal data with fixed effects for the linear variables of BWz, HCz, asymmetry, and categorical variable of cardiac lesion (SVP versus TGA) and random effects for participants allowing for separate intercepts for each participant. Asymmetry categories used were as defined in the methods section above. The outcome FA was log transformed due to non-normal distribution of FA secondary to outliers. An interaction term for each time invariant predictor (BWz, HCz, birth asymmetry, and cardiac lesion) by postmenstrual age at MRI was included in separate models to determine whether parameters of microstructural brain development evolved differently by these predictors. All statistical analyses were performed using Stata 16 (StataCorp, College, Texas).

## Results

A total of 173 infants with neonatal brain imaging data were included in the current study (TGA = 106; SVP = 67), of which 167 had data on anthropometric measures at birth. Of these, 69 infants underwent ND evaluation at 30 months of age. Of the 104 infants that did not undergo ND evaluation, 25 died prior to that time and 68 missed the 30 month visit or were lost to follow-up, while 11 infants were not yet 30 months of age.

**Fig. 1:**
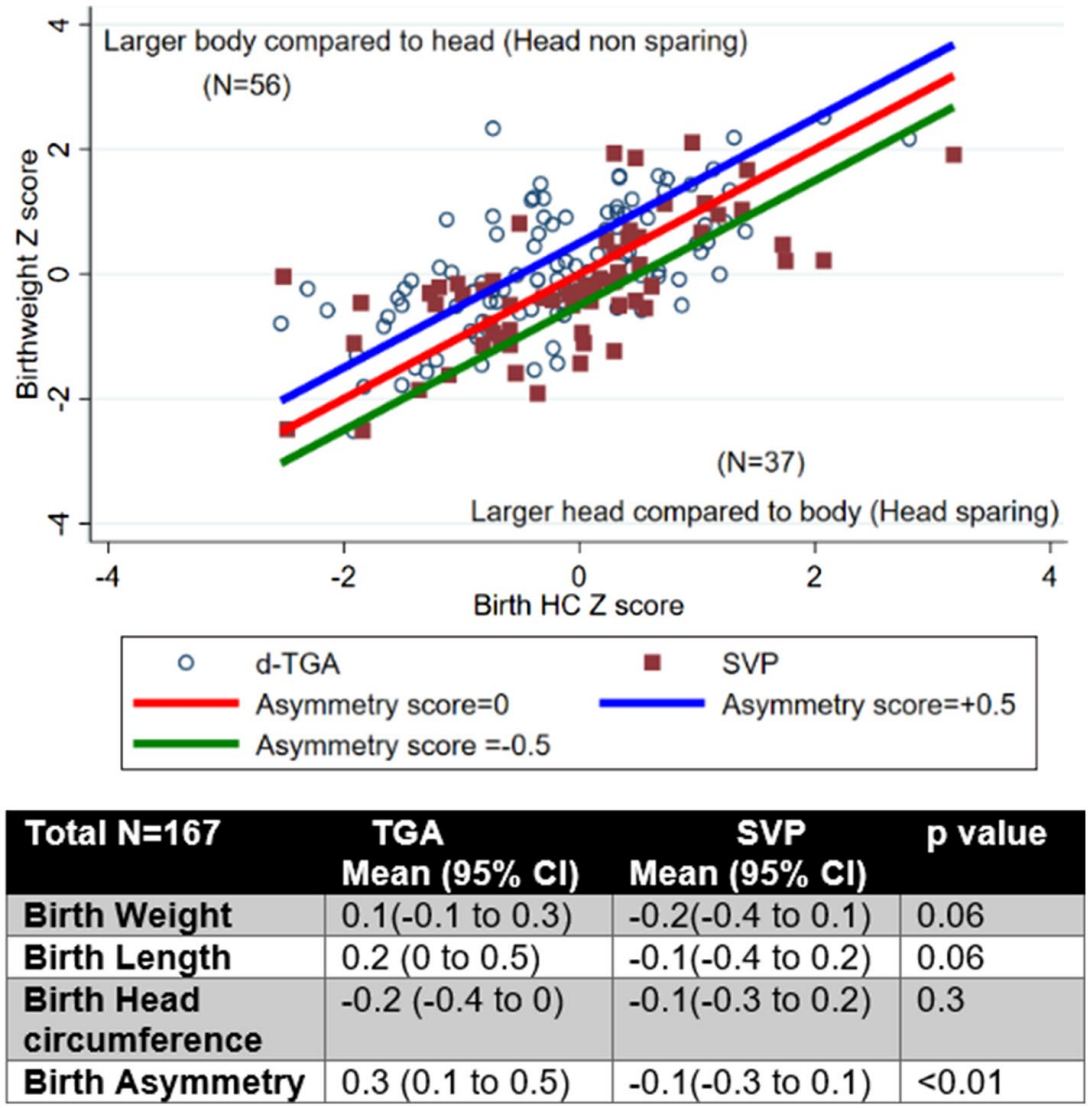
Relationship of size at birth by cardiac lesion type, the figure shows a plot of birth head circumference *Z* scores versus birth weight *Z* scores showing the relative weight to head circumference asymmetry of patients in the study-note that most infants are along the line of symmetry [birth asymmetry score of − 0.5 to + 0.5 (*N* = 74)], there is slightly higher proportion of the blue circles (TGA) above the line of symmetry. The table shows birth parameter *Z* scores by lesion type

Figure 1 shows a plot of BWz by HCz for each cardiac lesion and the distribution of asymmetry groups. Mean values of z-score measures at birth are listed by cardiac lesion. In general, measurements at birth were similar between SVP and TGA subjects, although TGA subjects tended to have smaller head circumference compared to weight (non-head sparing asymmetry) (mean BWz = 0.1 for TGA and − 0.2 for SVP, mean HCz = − 0.2 for TGA and − 0.1 for SVP).

A total of 150 infants had none or mild WMI on the pre-operative MRI and 23 had moderate-to-severe WMI. Baseline demographics of the cohort are listed in Table 1 by WMI severity on the pre-operative MRI. SVP subjects had a trend toward a higher frequency of moderate-to-severe WMI compared to TGA subjects (*p* = 0.06). No significant differences were noted in anthropometric measures at birth between those with and without moderate-to-severe WMI (Table 1). Similarly, there was no difference in BW, HC, length, or asymmetry Z scores by pre-operative BIS scores.

**Table 1:** Demographics of patients by white matter injury (WMI) severity (primary outcome) on pre-operative brain MRI

	WMI		*p*-value
	None/MILD (*n* = 150)	Mod/severe (*n* = 23)	
Race			
White non-Hispanic	85	15	
Hispanic	36	4	
Black	1	1	
Asian	14	2	
Other	14	1	0.5^±^
Cardiac group			
TGA, *N*(%)	96 (90.6)	10 (9.4)	
SVP, *N*(%)	54 (80.6)	13(19.4)	0.06^†^
GA birth weeks, mean 95%CI	39.2 (39,39.4)	39(38.5,39.5)	0.4^‡^
GA group			
Term, *N*(%)	93 (89.4)	11(10.6)	
Early term, *N* (%)	57 (82.6)	12(17.4)	0.2^†^
Site			
UBC	54 (90)	6(10)	
UCSF	96(85)	17(15)	0.4^†^
Birth weight *Z*-score Mean, 95% CI	0 (− 0.2, 0.2)	0 (− 0.3,0.3)	1^‡^
Head Circ *Z*-score Mean, 95% CI	− 0.2 (− 0.3,0)	− 0.1 (− 0.6,0.3)	0.9^‡^
Length *Z*-score Mean, 95% CI	0.1 (− 0.1,0.3)	0.3 (− 0.2,0.8)	0.4^‡^
Birth Asymmetry (BWZ-HCZ) Mean 95% CI	0.1 (0,0.3)	0 (− 0.3,0.5)	0.8^‡^

^‡^*T* test

^†^Pearson *χ*^2^ test

^±^Fisher’s exact test

FA measurements were only available for infants at the UCSF site (*n* = 103 infants). There was no association between mean white matter FA on pre-operative brain MRI and BW (*p* = 0.7), length (*p* = 0.8), HC (*p* = 0.5), or asymmetry (*p* = 0.7) *Z* scores.

**Fig. 2:**
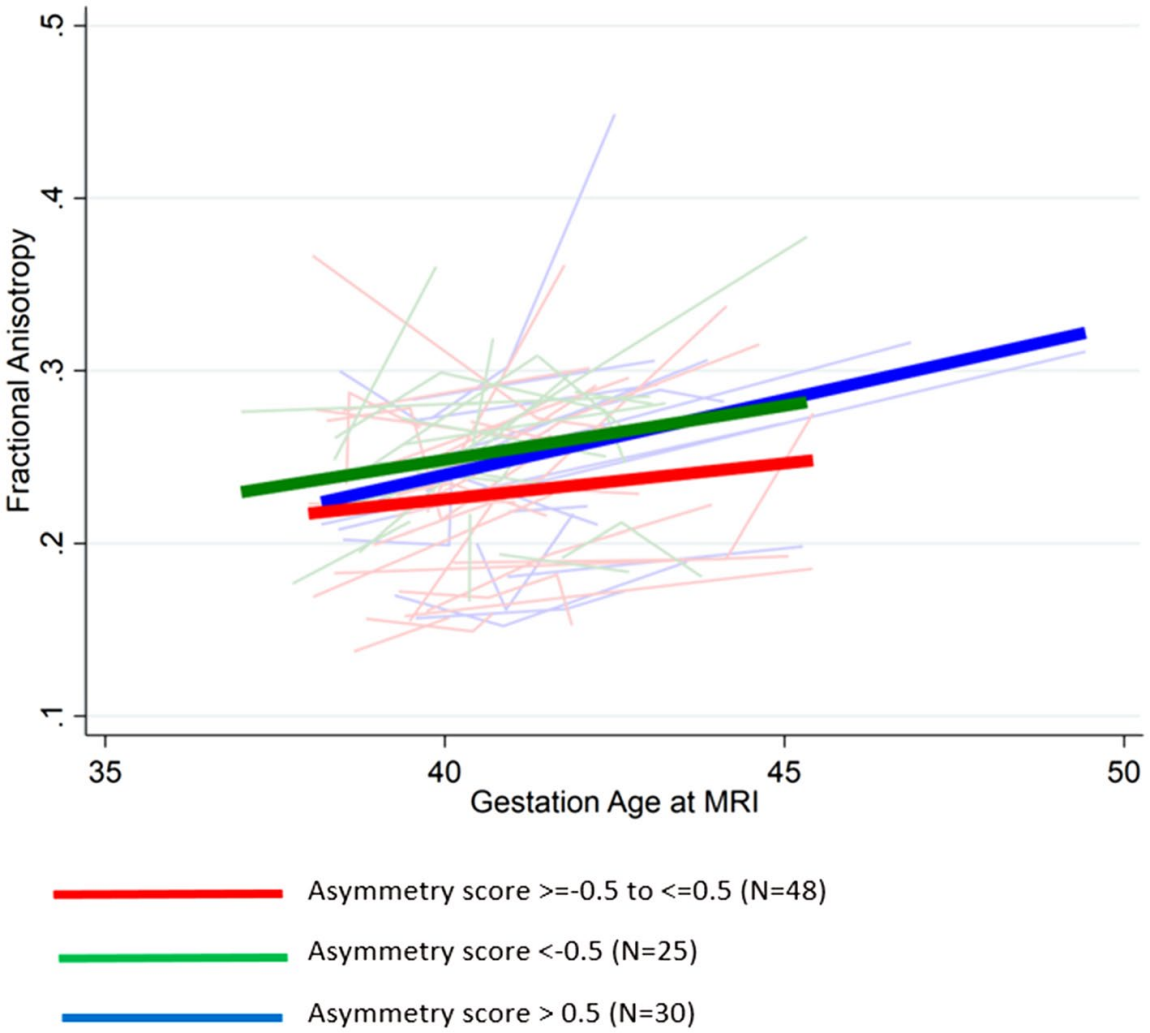
FA plotted against gestational age at MRI. Each line represents the change in FA between pre- and post-operative brain MRI for each infant. The 3 thick colored lines represent the regression lines for symmetric infants (red thick line) (Asymmetry score ≥ − 0.5 and < 0.5), head sparing asymmetry (green thick line) (Asymmetry score < − 0.5), and non-head sparing asymmetry (blue thick line) (Asymmetry score > 0.5). Note most infants showing increase in fractional anisotropy with gestation age at MRI reflecting brain maturation with time. No differences are apparent between the different ranges of asymmetry scores and this is confirmed by mixed model analysis

Measurements of size at birth were compared to the rate of change in FA from the pre- to post-operative MRI (Fig. 2). Overall, fractional anisotropy (FA) increased with gestational age at MRI. For every 1 week increase of gestational age at MRI, FA increased by 3.4% (95% CI 2.3, 4.4; *p* < 0.001). The rate of change in FA was assessed by the asymmetry category as defined above. FA increased by 2.8% per week of gestational age (95% CI 1.2, 4.4) for the symmetric group (BWZ-HCZ scores ≥ − 0.5 and ≤  + 0.5) while it increased by 2.9% per week of gestational age (95% CI 0.5, 5.4) for the head sparing group (BWZ-HCZ < − 0.5) and by 4.3% per week of gestational age (95% CI 2.4, 6.2) for the non-head sparing (BWZ-HCZ > 0.5) group. Compared to the symmetric group, there was no difference in the rate of change in FA in the head sparing group (*p* = 0.7) or in the non-head sparing group (*p* = 0.2).

Univariate linear regression analysis demonstrated no association between measures of size at birth and birth asymmetry with MDI or PDI at 30 months of age (Tables 2, 3). Among other variables assessed, only cardiac lesion and site were significantly associated with MDI or PDI at 30 months. More precisely, SVP patients had PDI scores on average 14.7 (95% CI − 23.1, − 6.2) points lower compared to TGA patients at 30 months.

**Table 2:** Linear regression analysis of the relationship between predictors with the Bayley II Mental Development Index (MDI) at 30 months

	Unadjusted analysis		*p*-value	Adjusted analysis	*p*-value
	Mean (SE)	Coefficient 95% CI		Coefficient 95% CI	
Cardiac Group					
TGA	95.1 (2)	Ref.		Ref.	
SVP	87.7 (4)	− 7.4 (− 15.7,0.9)	0.08	1.1 (− 7.7,9.8)^†^	0.8
GA birth (weeks)	–	1.2 (− 2.1,4.5)	0.5	1.3 (− 1.6,4.3)	0.4
GA birth group					
Term	93.8 (2.5)	Ref.		Ref.	
Early term	92.4 (2.4)	− 1.4 (− 8.9,6.1)	0.7	− 1.7 (− 8.5,5.1)	0.6
BW *z*-score	–	− 0.8 (− 4.4,2.9)	0.7	− 2.1 (− 5.5,1.2)	0.2
HC *z*-score	–	1 (− 2.5,4.5)	0.6	− 0.8 (− 4.1,2.5)	0.6
Length *z*-score	–	− 1.5 (− 5.1,2.1)	0.4	− 2.4(− 5.6,0.8)	0.1
Asymmetry BWz-HCz	–	− 2.7 (− 6.7,1.4)	0.2	− 2.2 (− 6,1.6)	0.3
Site					
UBC	98.9(2)	Ref.		Ref.	–
UCSF	84.5(2.7)	− 14.4(− 21, − 7.8)	< 0.001	− 14.9(− 22.6, − 7.1)*	< 0.001
Log (Hospital LOS)	–	− 1.8 (− 9.1,5.6)	0.6	− 3.1 (− 10.1,3.9)	0.4

The analysis was adjusted for site and cardiac group. Mean MDI are listed for categorical predictors

^†^Adjusted for site alone

*Adjusted for cardiac group/lesion alone

**Table 3:** Linear regression analysis of the relationship between predictors with the Bayley II Psychomotor Development Index (PDI) at 30 months

	Unadjusted analysis		*p*-value	Adjusted analysis	*p*-value
	Mean (SE)	Coefficient 95% CI		Coefficient 95% CI	
Cardiac group					
TGA	91.9 (2.1)	Ref.		Ref.	
SVP	77.2 (3.8)	− 14.7 (− 23.1,− 6.2)	0.001	− 8.3 (− 18.1,1.6) ^†^	0.1
GA birth (wks)	–	− 0.7 (− 4.3,3)	0.7	− 0.8 (− 4.1,2.5)	0.6
GA birth group					
Term	87.2 (2.5)	Ref.		Ref.	
Early term	90.1 (3.2)	2.9 (− 5.2,11)	0.5	3.6 (− 3.9,11)	0.3
BW *z*-score	–	1.7 (− 2.3,5.7)	0.4	0 (− 3.7,3.7)	1
HC *z*-score	–	1.32 (− 2.6,5.3)	0.5	0.4 (− 3.2,4.1)	0.8
Length *z*-score	–	0.1 (− 3.8,4)	1	− 1.3 (− 4.8,2.3)	0.5
Asymmetry BWz-HCz		− 0.2 (− 4.7,4.3)	0.9	− 1.1 (− 5.3,3)	0.6
Site					
UBC	93.9 (2.4)	Ref.		Ref.	–
UCSF	79.7 (2.6)	− 14.2 (− 21.4,− 6.9)	< 0.001	− 10.1(− 18.8,− 1.5)*	0.02
Log (Hospital LOS)	–	− 2.6 (− 10.2,5)	0.5	− 0.3 (− 7.6,7)	0.9

The analysis was adjusted for site and cardiac group. Mean PDI are listed for categorical predictors

^†^Adjusted for site alone

*Adjusted for cardiac group/lesion alone

## Discussion

In this study with a well characterized cohort of patients that underwent neonatal brain MRI and subsequent ND evaluation, we found that neither anthropometric measures at birth nor growth asymmetry were associated with risk of neonatal brain injury or with ND outcome. In contrast, cardiac lesion and site both demonstrate a significant association with ND outcome. Our findings further add to the body of literature on risk factors associated with neurodevelopment in the CHD population and the association of fetal growth patterns with ND outcomes.

Our rationale to study somatic size in newborns with CHD is related to the observations in previous studies that newborns with CHD have lower body weight and smaller HC compared to infants without CHD [8]. A large analysis of infants from the Danish nationwide cohort has recently shown that infants with hypoplastic left heart syndrome and other single ventricle defects have smaller HC (mean adjusted *Z* score − 0.39 95% CI − 0.21, − 0.58) and lower body weight (mean adjusted *Z* scores − 0.38 95% CI − 0.19, − 0.57) [8] though are typically symmetrical in size. Conversely, infants with TGA have smaller HC (mean adjusted *Z* score − 0.29 95% CI − 0.16, − 0.43) but normal body weight (mean adjusted *Z* score − 0.03 95% CI − 0.17, 0.1) consistent with growth asymmetry. Aberrant fetal circulation in the setting of TGA can explain this growth asymmetry whereby highly oxygenated blood passes through the foramen ovale to the left ventricle and pulmonary outflow tract while deoxygenated blood preferentially supplies the aorta and cerebral vasculature leading to decreased oxygen and nutrient delivery to the brain [12]. Fetuses with SVP, particularly those with hypoplastic left heart syndrome also have decreased oxygen and nutrient delivery to the brain though, since there is complete intracardiac mixing, a discrepancy of blood flow to the upper and lower bodies would not be expected. In turn, both fetal and neonatal brain MRI studies have shown that complex CHD including SVP and TGA is associated with delayed brain development beginning in utero, including smaller total brain volume [31, 32]. Importantly, perinatal impairments in brain growth appear to affect subsequent brain growth trajectories in CHD [33]. Thus, we hypothesized that somatic growth in utero, particularly growth asymmetry in TGA, would be a proxy for measures of brain health such as acquired brain injury and/or neurodevelopmental outcomes. Indeed, prior studies have shown that measures of somatic growth in infancy are associated with ND outcomes in SVP [5]. Similar to prior studies, in our study TGA newborns exhibited asymmetry with a smaller head circumference relative to body weight, whereas newborns with SVP were more likely to be symmetrical. However, we did not find an association between somatic size at birth, including degree of asymmetry, with acquired brain injury and ND outcomes. This finding is consistent with a recent secondary analysis of the infants enrolled in the SVR trial reported by Miller et al. [7] which did not show an association between weight for gestational age and growth asymmetry with developmental outcomes at 6 years for patients with hypoplastic left heart syndrome [7]. Our findings are in contrast to those reported by Williams et al. where asymmetry as measured by fetal head circumference to abdominal circumference ratio was negatively associated with ND outcomes [25]. However, the analysis was based on a heterogenous group of patients with varying forms of CHD which may confound the relationship between fetal growth parameters and ND outcomes.

Our findings are in contrast to other patient populations such as growth restriction in term and early term infants where “brain-sparing” fetal physiology and a smaller head circumference at birth correlates with poorer ND outcomes [15]. In this population it has been shown that infants with evidence of placental dysfunction on pathology or by Doppler studies on fetal ultrasound have significantly worse ND outcomes than those without [34]. Placental insufficiency in this population leads to redistribution of cerebral blood flow with preferential flow to the deep gray matter in the basal ganglia as demonstrated by studies using fractional moving blood volume [35–37]. It is postulated that this redistribution occurs at the expense of blood flow to the frontal and parietal white matter leading to decreased growth and increased susceptibility of white matter areas to hypoxic injury. In contrast, the mechanism of asymmetry in CHD and, in particular, TGA, is likely different to that seen in the growth-restricted fetus related to the timing of the insult. Placental insufficiency and subsequent fetal hypoxia in the case of growth-restricted fetuses is more likely to occur in the second and third trimester while the formation of the fetal heart and establishment of an aberrant fetal circulation in the case of TGA is complete within the first trimester. Despite the asymmetry observed in TGA, fetuses with TGA have less profound alterations in cerebral blood flow (MCA) compared to SVP and growth-restricted fetuses [10, 38–40]. In fact, some studies have failed to show any significant alterations in MCA dopplers in TGA [38, 40]. This suggests that, in contrast to fetal growth restriction, fetal cerebrovascular changes and subsequent ND outcomes may be unrelated to the asymmetry observed in TGA. Indeed, this is consistent with the lack of association between asymmetry, brain injury, and ND outcomes seen in our study cohort. The findings of our study suggest that, unlike in other high-risk infant populations, postnatal markers of fetal growth may not be helpful in prognosticating ND outcomes in infants with CHD. This finding underscores the importance of using other methods of obtaining neuro-prognostic data such as brain MRI in fetuses and neonates with CHD.

Recent studies have suggested that ND impairment in CHD can be explained by additional pathology including placental insufficiency or genetic anomalies rather than as an effect of altered hemodynamics alone [41, 42]. Similar to growth restriction, ND outcomes have been found to be significantly worse in the children who had placental pathology when compared to those with isolated CHD [43]. Placental and genetic abnormalities can co-occur in CHD pregnancies [41, 44–46], significantly contributing to ND outcomes in this patient population.

Interestingly, we found that the strongest predictors of ND outcomes were site and cardiac lesion. This is in line with literature suggesting that medical complexity has a more significant impact on ND outcomes [47–50]. The association of lesion with ND outcomes is in line with our previous finding that infants with SVP have slower peri-operative brain growth compared to TGA infants [51], likely related to the fact that they have ongoing abnormal cardiovascular physiology whereas in TGA there is restoration of normal physiology after the arterial switch operation. A recent study has shown reduced placental cell activity and nutrient transport in SVP (particularly hypoplastic left heart syndrome), when compared to TGA [52]. It is conceivable that such differences in placental function may explain some of the differences in pre-operative brain injury and ND outcomes between the 2 groups. Infants with SVP also undergo a greater number of surgeries and have longer overall length of stay in the hospital than TGA. Hospital length of stay is a known independent predictor of ND outcomes in the CHD population [13, 47, 49, 50, 53]. Although infants with TGA appear to have minimal ND impairments in our study, other longitudinal studies have shown subtle yet pervasive differences in ND including lower academic, cognitive, memory, and attention performance scores, as well as increased social difficulties than those of test normative populations and thus would benefit from long-term ND surveillance and follow-up [53].

The finding that site played a significant role in ND outcomes is also not surprising as there are differences in socioeconomic status, postnatal environment, and testing operators between the 2 sites. Alternatively, given the higher scores seen in the Canadian cohort, other factors like attrition bias may be playing a role in this difference. Literature on preterm children has suggested an attrition bias in the rate of ND impairments that varies by country [54]. When comparing Canada with the USA, attrition rates were much lower in Canada and this was associated with a lower rate of ND impairments at 18 to 24 months in Canada. This can be secondary to different health care systems and possibly easier accessibility to screening and early intervention services in Canada. In our study, the rate of follow-up at 30 months was lower in the UCSF cohort as compared with the Canadian cohort, which may have influenced the PDI and MDI scores.

In conclusion, our study shows that infants with SVP are symmetrical while TGA is associated with non-head sparing asymmetry, supporting the role of circulatory physiology in growth and symmetry as shown in prior studies. In contrast to other patient populations such as fetal growth restriction, postnatal markers of fetal growth may not be helpful in prognosticating ND outcomes in SVP and TGA infants underscoring the importance of other methods (such as brain imaging) of obtaining neuroprognostic data in these neonates. Lesion type (SVP) plays a more significant role in ultimate ND outcomes including risk of neonatal brain injury. With increasing data to suggest a high rate of placental abnormalities in pregnancies affected by CHD, future studies can be focused on how the maternal–fetal environment in the setting of CHD may influence placental function and ultimately ND outcomes.

## Supplementary Information

Below is the link to the electronic supplementary material.

Supplementary file1 (DOCX 13 KB)

## Data Availability

The data that support the findings of this study are available from the corresponding author upon reasonable request.

## Abbreviations

BI: Brain injury
NDO: Neurodevelopmental outcomes
BW: Birth weight
HC: Head circumference
WMI: White matter injury
SVP: Single ventricle physiology
TGA: D-Transposition of great arteries
MRI: Magnetic resonance imaging
CHD: Congenital heart disease
ND: Neurodevelopment
UCSF: University of California San Francisco Benioff Children’s Hospital
UBC: University of British Columbia
FA: Fractional anisotropy
BSID-II: Bayley Scales of Infant Development-II
PDI: Psychomotor Development Index
MDI: Mental Development Index

## Code Availability

Not applicable to this study.
