# Supplementary material for: The Effect of Size and Asymmetry at Birth on Brain Injury and Neurodevelopmental Outcomes in Congenital Heart Disease
Source: Pediatr Cardiol. 2021 Dec 1;43(4):868–77. doi: 10.1007/s00246-021-02798-5 (PMC9005428; doi:10.1007/s00246-021-02798-5)
Supplement: Supplementary file 1 — Supplementary file1 (DOCX 13 KB) [file 246_2021_2798_MOESM1_ESM.docx]

# Supplement

**Detailed MRI methods:**

MRI studies at UCSF were performed with pharmacologic sedation, as needed, on a 1.5 or 3 Tesla system (GE Healthcare Signa Echo-speed; Waukesha, WI, USA) using GE EXCITE 1.5T/3T software and included: 4-mm-thickness T1-weighted sagittal and axial spin echo, 4-mm-thickness dual-echo T2-weighted spin echo, 1.5-mm-thickness coronal or sagittal volumetric three-dimensional gradient echo with radiofrequency spoiling images, and a diffusion tensor sequence (repetition time: 7000ms; echo time: 99.5ms; 3-mm section thickness; no gap; three repetitions per image, with 18x36cm field of view and 128x256 acquisition matrix), acquiring axial images through the whole brain with an in-plane resolution of 1.4x1.4mm^2^. Seven images per axial section were acquired, including a T2-weighted reference image (b = 0 s/mm^2^) and 6-15 diffusion-weighted images (b = 700 s/mm^2^) in noncollinear gradient directions. At UBC, MRI studies were carried out without pharmacologic sedation on a Siemens 1.5 Tesla Avanto (Siemens AG, Healthcare Sector, Erlange, Germany) using a VB 13A software (Siemens AG, Healthcare Sector) and included 3-dimensional coronal volumetric T1-weighted images (TR, 36 msec; TE, 9.2 msec; field of view, 200 mm; slice thickness, 1 mm; section gap, 0) and axial fast spin-echo T2-weighted images (TR, 4610 msec; TE, 107 msec; field of view 160 mm; slice thickness, 4 mm; section gap, 0.2 mm). A diffusion tensor sequence was acquired with a multirepetition single-shot echo planar sequence with twelve gradient directions (TR, 4900ms; TE, 104ms; field of view, 160 mm; slice thickness, 3mm; section gap, 0), three averages of two diffusion weightings of 700 and 1000 seconds per square millimeter (b value) and an image without diffusion weighting, with an in-plane resolution of 1.3 mm.
